# Supplementary material for: Can We Reliably Detect Respiratory Diseases through Precision Farming? A Systematic Review
Source: Animals (Basel). 2023 Apr 6;13(7):1273. doi: 10.3390/ani13071273 (PMC10093556; doi:10.3390/ani13071273)
Supplement: Supplementary file 1 [file animals-13-01273-s001.zip › animals-2142184-supplementary 2.pdf]

Supplementary Table S2. Data gathered from articles about PLF technologies for swine, bovine and poultry productions. It is presented data about the production stage, production system, and aim of the production for each study included in the review.

Species	Author	Stage	Production system	Aim of the production
Swine	[30]	Fattening	Experimental setup	Meat
	[31]	Fattening	Experimental setup	Meat
	[32]	Finishing	Conventional indoor housing	Meat
	[33]	Finishing	Conventional indoor housing	Meat
	[34]	Fattening	Experimental setup	Meat
	[35]	Fattening	Conventional indoor housing	Meat
	[36]	Finishing	Conventional indoor housing	Meat
	[37]	Fattening	Conventional indoor housing	Meat
	[15]	Fattening	Conventional indoor housing	Meat
	[38]	Not possible to retrieve	Experimental setup	Meat
	[39]	Fattening	Conventional indoor housing	Meat
	[40]	Fattening	Conventional indoor housing	Meat
	[41]	Fattening	Conventional indoor housing	Meat
Poultry	[42]	14 day old	Experimental setup	Unclear
	[43]	Newborn chicks	Experimental setup	Unclear
	[14]	7 day old	Experimental setup	Meat
	[44]	N/A	Experimental setup	Unclear
	[45]	30 day old	Dark House	Meat
	[46]	15 day old	Experimental setup	Unclear
Bovine	[47]	Calves	Auction	Beef
	[13]	Calves	Indoor group housing	Dairy
	[48]	Calves	Indoor group housing	Dairy
	[16]	Calves	Indoor group housing	Dairy
